# Effects of Cr Substitution on Negative Thermal Expansion and Magnetic Properties of Antiperovskite Ga_1−*x*_Cr_*x*_N_0.83_Mn_3_ Compounds

**DOI:** 10.3389/fchem.2018.00075

**Published:** 2018-03-21

**Authors:** Xinge Guo, Peng Tong, Jianchao Lin, Cheng Yang, Kui Zhang, Shuai Lin, Wenhai Song, Yuping Sun

**Affiliations:** ^1^School of Science, Hebei University of Science and Technology, Shijiazhuang, China; ^2^Key Laboratory of Materials Physics, Institute of Solid State Physics, Chinese Academy of Sciences, Hefei, China; ^3^High Magnetic Field Laboratory, Chinese Academy of Sciences, Hefei, China; ^4^Collaborative Innovation Center of Advanced Microstructures, Nanjing University, Nanjing, China

**Keywords:** negative thermal expansion, antiferromagnetic order, specific heat, antiperovskite compounds, Cr substitution

## Abstract

Negative thermal expansion (NTE) and magnetic properties were investigated for antiperovskite Ga_1−*x*_Cr_*x*_N_0.83_Mn_3_ compounds. As *x* increases, the temperature span (Δ*T*) of NTE related with Γ^5g^ antiferromagnetic (AFM) order is expanded and shifted to lower temperatures. At *x* = 0.1, NTE happens between 256 and 318 K (Δ*T* = 62 K) with an average linear coefficient of thermal expansion, α_*L*_ = −46 ppm/K. The Δ*T* is expanded to 81 K (151–232 K) in *x* = 0.2 with α_*L*_ = −22.6 ppm/K. Finally, NTE is no longer visible for *x* ≥ 0.3. Ferromagnetic order is introduced by Cr doping and continuously strengthened with increasing *x*, which may impede the AFM ordering and thus account for the broadening of NTE temperature window. Moreover, our specific heat measurement suggests the electronic density of states at the Fermi level is enhanced upon Cr doping, which favors the FM order rather than the AFM one.

## Introduction

Negative thermal expansion (NTE) materials, which contract upon heating, have received great attentions recently (Mary et al., 1996; Takenaka and Takagi, 2005; Goodwin et al., 2008; Long et al., 2009; Greve et al., 2010; Azuma et al., 2011; Yamada et al., 2011; Huang et al., 2013; Panda et al., 2014; Zhao et al., 2015). From the view point of applications, NTE materials can be used as fillers for compensating and controlling the positive thermal expansion (PTE) of normal materials by forming composites (Romao et al., 2003; Chen et al., 2015). NTE has been observed in many materials due to different mechanisms, including flexible framework in crystal structure (Mary et al., 1996; Goodwin et al., 2008; Greve et al., 2010; Ge et al., 2016; Hu et al., 2016; Jiang et al., 2016), ferroelectricity (Xing et al., 2003; Chen et al., 2013), charge transformation (Long et al., 2009; Azuma et al., 2011; Yamada et al., 2011), magnetovolume effect (MVE) (Takenaka and Takagi, 2005; Huang et al., 2013; Li et al., 2015, 2016), and martensitic transformation (Zhao et al., 2015; Lin et al., 2016). Among them, the NTE related with MVE in antiperovskite manganese nitrides ANMn_3_ (A: transition metal or semiconducting elements) has been extensively studied because of the large and isotropic NTE with tunable linear coefficient of thermal expansion (α_*L*_), good mechanical properties (large Young's modulus and hardness) and thermal/electrical conductivities (Takenaka and Takagi, 2005; Sun et al., 2007; Huang et al., 2008; Song et al., 2011; Tong et al., 2013a,b; Tan et al., 2014).

Large lattice volume contraction of a few percent at the antiferromagnetic (AFM) to paramagnetic (PM) phase transition due to MVE has been reported in antiperovskite manganese nitrides decades ago (Fruchart and Bertaut, 1978). However, due to the limited temperature window (a few K) of MVE, these materials cannot be practically used as PTE compensators. In 2005, Takenaka firstly reported the broadening of MVE window in Cu_1−*x*_Ge_*x*_NMn_3_ (Takenaka and Takagi, 2005). From then on, many studies reported the NTE properties in ANMn_3_ (A = Zn, Ga, Ag, and Cu) by substituting A with non-magnetic elements, such as Ge, Sn, Si (Sun et al., 2007, 2010a,b; Huang et al., 2008; Takenaka et al., 2008; Dai et al., 2014). Neutron diffraction studies indicated that the pronounced MVE occurs due to the ordering of the non-collinear triangular Γ^5g^ AFM spin configuration, and the non-magnetic element doping slows down the ordering of Γ^5g^ AFM phase (Iikubo et al., 2008a; Song et al., 2011; Deng et al., 2015a,b). Local structure measured via the neutron pair distribution function (PDF) (Iikubo et al., 2008b; Tong et al., 2013a) and x-ray absorption fine structure measurements (Matsuno et al., 2009) suggested a strong relation between the broadening of AFM transition and the local lattice distortions, though a detailed mechanism is still under debate (Tong et al., 2013a). Very recently, we found that by partially replacing A in ANMn_3_ (i.e., GaN_0.8_Mn_3_, AgNMn_3_) with Mn, the MVE window was expanded as well (Guo et al., 2015; Lin et al., 2015), while local structural distortion was not observed (Guo et al., 2015). For example, in Ga_1−*x*_Mn_*x*_N_0.8_Mn_3_ the Δ*T* of NTE reaches 54 K (between 255 and 309 K, α_*L*_ = −42 ppm/K) and 73 K (between 206 and 279 K, α_*L*_ = −25 ppm/K) for *x* = 0.25 and 0.3, respectively (Guo et al., 2015). Large NTE with α_*L*_ ~ −20 ppm/K at cryogenic temperatures (below 120 K) was achieved in (Ga_0.7_Cu_0.3_)_1−*x*_Mn_*x*_NMn_3_ with *x* = 0.25 and 0.3 (Guo et al., 2017). In those Mn-doped compounds, in addition to the AFM order that gives rise to the large volume change, the coexisting FM order was demonstrated to impede the growth of the AFM order and thus cause the broadened Δ*T* of lattice contraction (Guo et al., 2015, 2017; Lin et al., 2015). It is interesting to check whether other 3d elements can tune the NTE of ANMn_3_ as the Mn does.

Here, we report influences of Cr substitution for Ga on thermal expansion and magnetic properties of MVE-compound GaN_0.83_Mn_3_. GaN_0.83_Mn_3_ is AFM below *T*_N_ ~ 360 K (Kasugai et al., 2012). Upon substituting Cr for Ga, the AFM ground is quickly suppressed. Meanwhile, FM order is introduced and increasingly enhanced with increasing Cr doping level. Accompanying with the suppression of AFM state, the sharp MVE of the parent compound is quickly moved to lower temperatures and the related temperature range is widened. A quite large NTE temperature window of 81 K (151–232 K) with a considerably large average α_*L*_ ~ −22.6 ppm/K was observed in *x* = 0.2. The emergence of FM order can be attributed to the increasing electronic density of states (DOS) at the Fermi energy (E_F_) as indicated by the increased electronic contribution to the specific heat at low temperatures.

## Experimental

Polycrystalline samples Ga_1−*x*_Cr_*x*_N_0.83_Mn_3_ (*x* = 0, 0.1, 0.2, 0.3, 0.4) were prepared by direct solid state reaction with Ga ingot (4N), Cr (3N), Mn (4N), and self-made Mn_2_N powders. The starting materials were mixed in the desired proportions, sealed in evacuated quartz tubes (10^−3^ Pa) and then annealed at 873–973 K for 5 days. After quenching the tubes to room temperature, the products were pulverized, mixed, pressed into pellets, and annealed again at 1,073–1,173 K for extra 8 days. The final samples were checked by X-ray diffraction (XRD) on a Bruker X-ray diffractometer (D8 Advance) with Cu Kα radiations at room temperature. The magnetization measurements were performed on a Superconducting Quantum Interference Device Magnetometer (SQUID, Quantum Design). By using a strain gauge, linear thermal expansion Δ*L*/*L* was measured on a Physical Property Measurement System (PPMS, Quantum Design; Lin et al., 2015). On the same PPMS system, specific heat was measured for *x* = 0 and 0.2 compounds.

## Results and discussion

**Figure 1:**
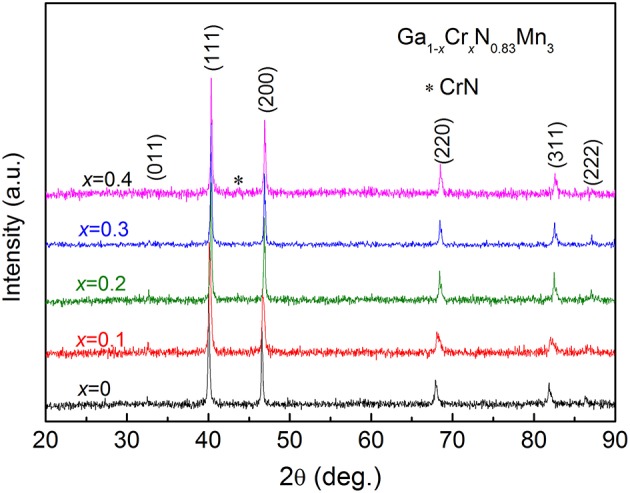
X-ray diffractions at room temperature for Ga_1−*x*_Cr_*x*_N_0.83_Mn_3_ (*x* = 0, 0.1, 0.2, 0.3, 0.4). The asterisk marks the diffractions from CrN.

**Figure 2:**
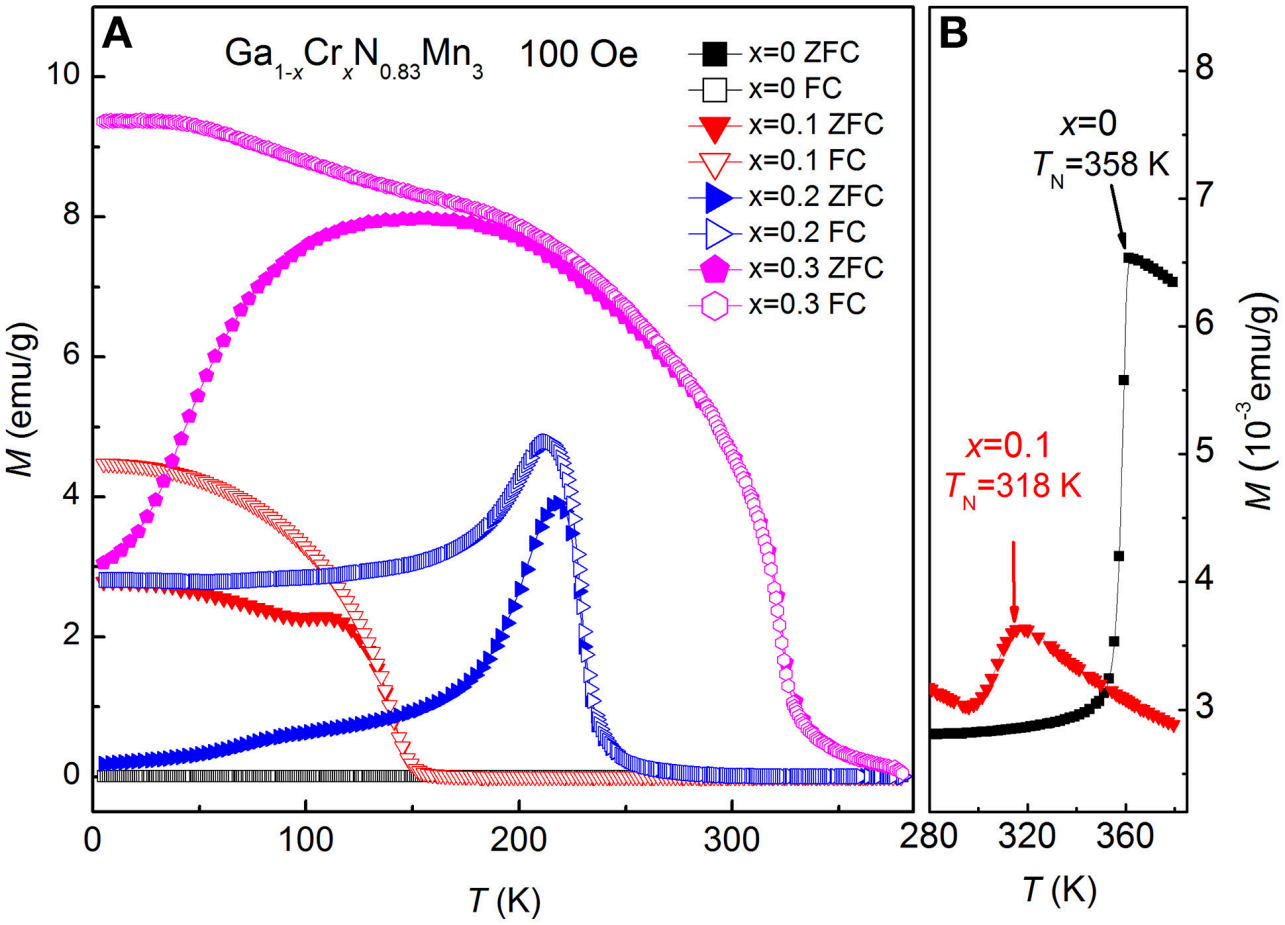
**(A)** The magnetization *M*(*T*) measured at both zero-field-cooling (ZFC) and field-cooling (FC) modes for Ga_1−*x*_Cr_*x*_N_0.83_Mn_3_ (*x* = 0, 0.1, 0.2, 0.3). **(B)** shows an enlargement of the high-temperature ZFC data for *x* = 0 and 0.1, where the antiferromagnetic to paramagnetic transition at *T*_N_ is marked in each curve.

**Figure 3:**
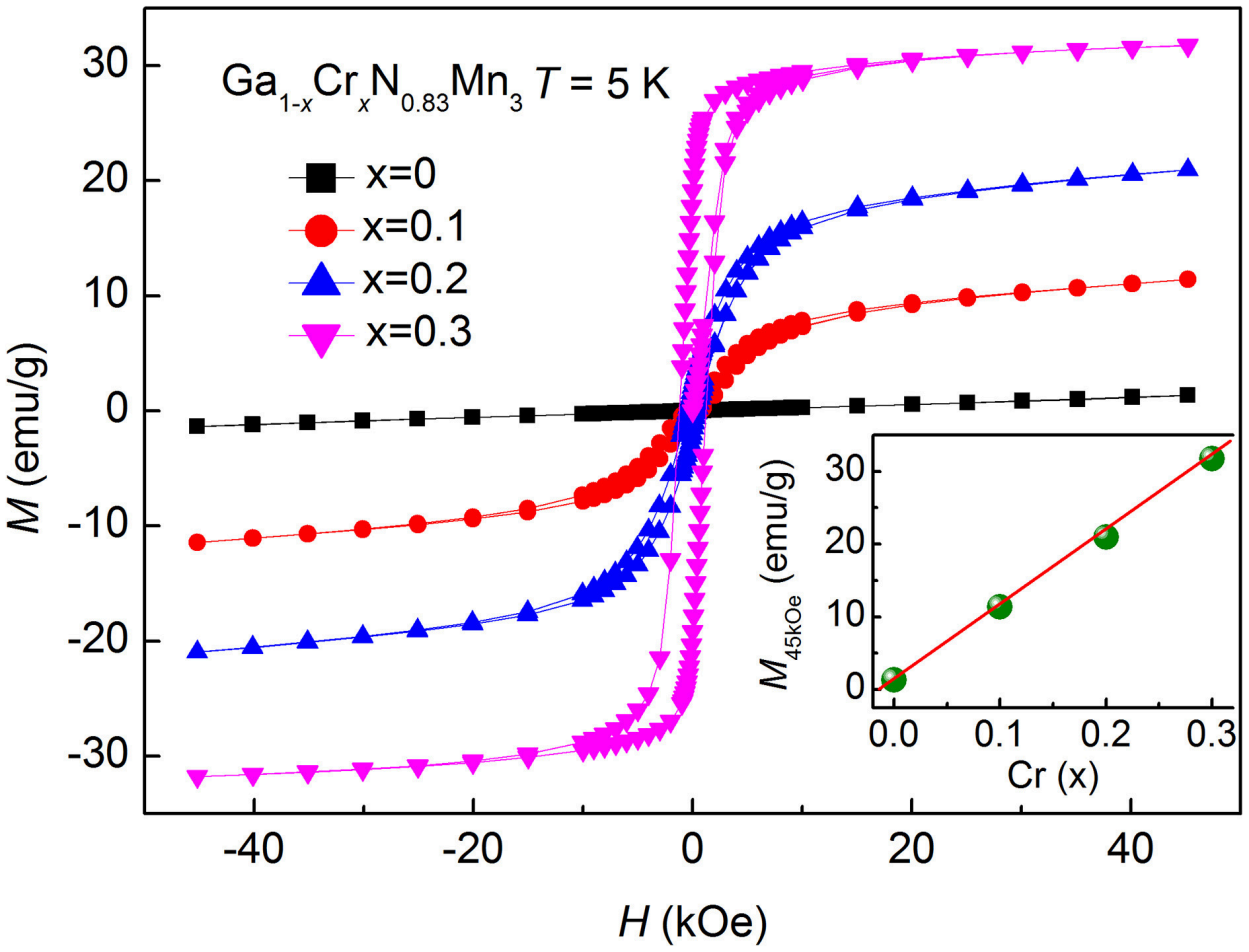
The isothermal magnetization *M*(*H*) loops at 5K for Ga_1−*x*_Cr_*x*_N_0.83_Mn_3_ (*x* = 0, 0.1, 0.2, 0.3) measured between −45 and 45 kOe. Inset shows the magnetization at 45 kOe, *M*_45kOe_, as a function of Cr content (*x*).

Figure 1 shows the room-temperature XRD patterns for Ga_1−*x*_Cr_*x*_N_0.83_Mn_3_ (*x* = 0, 0.1, 0.2, 0.3, 0.4) samples. All the samples are single-phase with a typical cubic antiperovskite structure (space group: Pm-3m), except for a very small amount of CrN detected in *x* = 0.4. The (111) peak shifts toward higher angles as *x* increases, which indicates the decrease of lattice constant with the increase of Cr content. Figure 2A presents the temperature dependent magnetization *M*(*T*) of Ga_1−*x*_Cr_*x*_N_0.83_Mn_3_ (0 ≤ *x* ≤ 0.3) measured at *H* = 100 Oe under both zero-field-cooling (ZFC) and field-cooling (FC) modes. As shown in Figure 2B, there is a kink at 358 K for *x* = 0, indicating an AFM to PM transition as often observed in antiperovskite manganese nitrides. This value agrees well with the Neel temperature (*T*_N_) of GaN_0.83_Mn_3_ reported previously (Kasugai et al., 2012). When *x* = 0.1, *T*_N_ is decreased to 318 K. In slightly Mn-doped Ga_1−*x*_Mn_*x*_N_0.8_Mn_3_ (Guo et al., 2015), (Ga_0.7_Cu_0.3_)_1−*x*_Mn_*x*_N_0.8_Mn_3_ (Guo et al., 2017), and Ag_1−*x*_Mn_*x*_NMn_3_ (Lin et al., 2015), the *M*(*T*)s are featured by a clear peak in the ZFC curves, while the related FC *M*(*T*)s show a FM-like transition. This behavior was verified as a glassy transition (Guo et al., 2015, 2017; Lin et al., 2015). In contrast, for *x* = 0.1 Cr doped sample, FM-like transition was observed at around 120 K in both ZFC and FC *M*(*T*) curves with an obvious divergence between them at lower temperatures. The absence of peak in ZFC *M*(*T*) curve is indicative of the emergence of long range FM order below 120 K. For *x* = 0.2, the FM-like transition is increased to 230 K. The kink on ZFC *M*(*T*) referring to *T*_N_ is no longer visible. Instead, a drop of magnetization happens at 210 K in both ZFC and FC *M*(*T*) curves, similar to that observed in Ga_1−*x*_Mn_*x*_N_0.8_Mn_3_ with *x* = 0.3 (Guo et al., 2015). The *M*(*T*) curves for *x* = 0.3 display a FM transition at 322 K, though the FC curve deviates from the ZFC one at low temperatures. Figure 3 shows the isothermal hysteresis loop *M*(*H*)s at 5 K for *x* = 0–0.3 samples. The magnetization at 45 kOe (*M*_45kOe_) increases quickly and linearly with increasing *x* (inset of Figure 3). At the same time the slopes of *M*(*H*) curves at high magnetic fields become smaller as *x* increases, indicating the FM component is enhanced at the expense of AFM component. For *x* = 0.3, a FM ground state is established.

**Figure 4:**
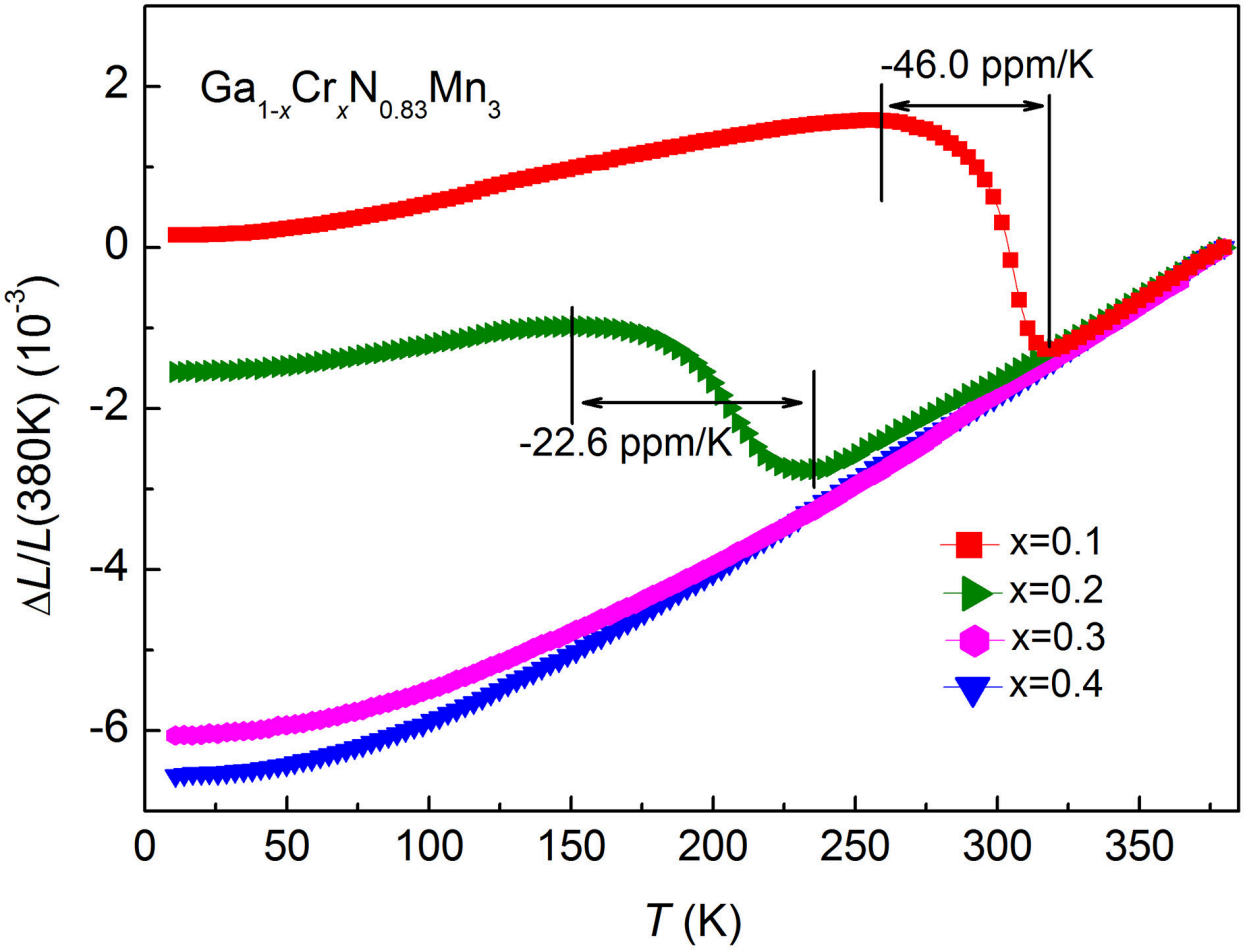
Linear thermal expansion Δ*L*/*L* (380 K) for Ga_1−*x*_Cr_*x*_N_0.83_Mn_3_ (*x* = 0.1, 0.2, 0.3 and 0.4). The temperature range of negative thermal expansion and the related average linear coefficient of thermal expansion are marked for *x* = 0.1 and 0.2.

Figure 4 shows the linear thermal expansion Δ*L*/*L* (380 K) for Ga_1−*x*_Cr_*x*_N_0.83_Mn_3_ (0.1 ≤ *x* ≤ 0.4). Because of the large volume change at *T*_N_ which is above room temperature, the as-prepared GaN_0.83_Mn_3_ sample was brittle and thus not subjected to the strain gauge measurement. As shown in Figure 4, at *x* = 0.1, the lattice undergoes a continuous shrinkage upon heating between 256 and 318 K (Δ*T* = 62 K) with an average α_*L*_ = −46 ppm/K. The onset temperature of NTE region is consistent with the broad AFM transition shown in Figure 2B. For *x* = 0.2, The NTE temperature window shifts to 151–232 K (Δ*T* = 81 K), and the corresponding average α_*L*_ is about ~ −22.6 ppm/K. The lattice contraction coincides well with the drop of magnetization displayed in both ZFC and FC *M*(*T*)s as shown in Figure 2A. When *x* is further increased (≥0.3), no NTE was observed down to 5 K.

Among the many ordered spin configurations, the Γ^5g^-type AFM one is special because it adopts a larger lattice volume relative to the PM or FM state, which is considered as the prerequisite for the showing up of NTE (Takenaka et al., 2014). The Γ^5g^-type AFM order is the ground state below *T*_N_ for the *x* < 0.2 compounds (Kasugai et al., 2012). Most likely, this particular AFM order is involved below 210 K in *x* = 0.2 sample, as manifested by the drop of the magnetization shown in Figure 2A. Upon doping with Cr, the FM order emergences and becomes increasingly strong with *x*, as revealed by enhanced *T*_C_ and the low-temperature magnetization. The strengthened FM phase would impede the growth and propagation of AFM order upon cooling probably via the magnetically coupled AFM/FM interfaces (Guo et al., 2015). When *x* is increased to 0.3, the FM phase is overwhelmingly strong so that the MVE associated with the AFM ordering is no longer able to influence the overall thermal expansion. As a result, the *x* = 0.3 compound displays a normal PTE.

The parent compound of Ga_1−*x*_Mn_*x*_N_0.8_Mn_3_ is very close to that of the current solid solutions in terms of the chemical composition and the value of *T*_N_. However, Cr doping is more effective in disturbing the AFM order and consequently in expanding the temperature range of lattice contraction relative to Mn doping. For example, with 20% Cr doping the Δ*T* of NTE is about 80 K, which is even larger than that of 30% Mn doped sample (Δ*T* = 73 K; Guo et al., 2015). As shown in the inset of Figure 3, *M*_45kOe_ at 5 K increases linearly with Cr doping level and reaches 31.8 emu/g for *x* = 0.3. But, for Ga_1−*x*_Mn_*x*_N_0.8_Mn_3_ the value of M_45kOe_ at 5 K shows a tendency toward saturation with increasing *x*, and the related value for *x* = 0.3 is only 22.3 emu/g (Guo et al., 2015). Such a difference indicates the more rapid strengthening of FM order in Cr-doped compounds than in Mn-doped ones. So the AFM phase in Cr-doped sample experienced a stronger impendence from the more rapidly developing FM order, leading to a wider NTE window relative to Mn-doped compounds at the same doping level.

**Figure 5:**
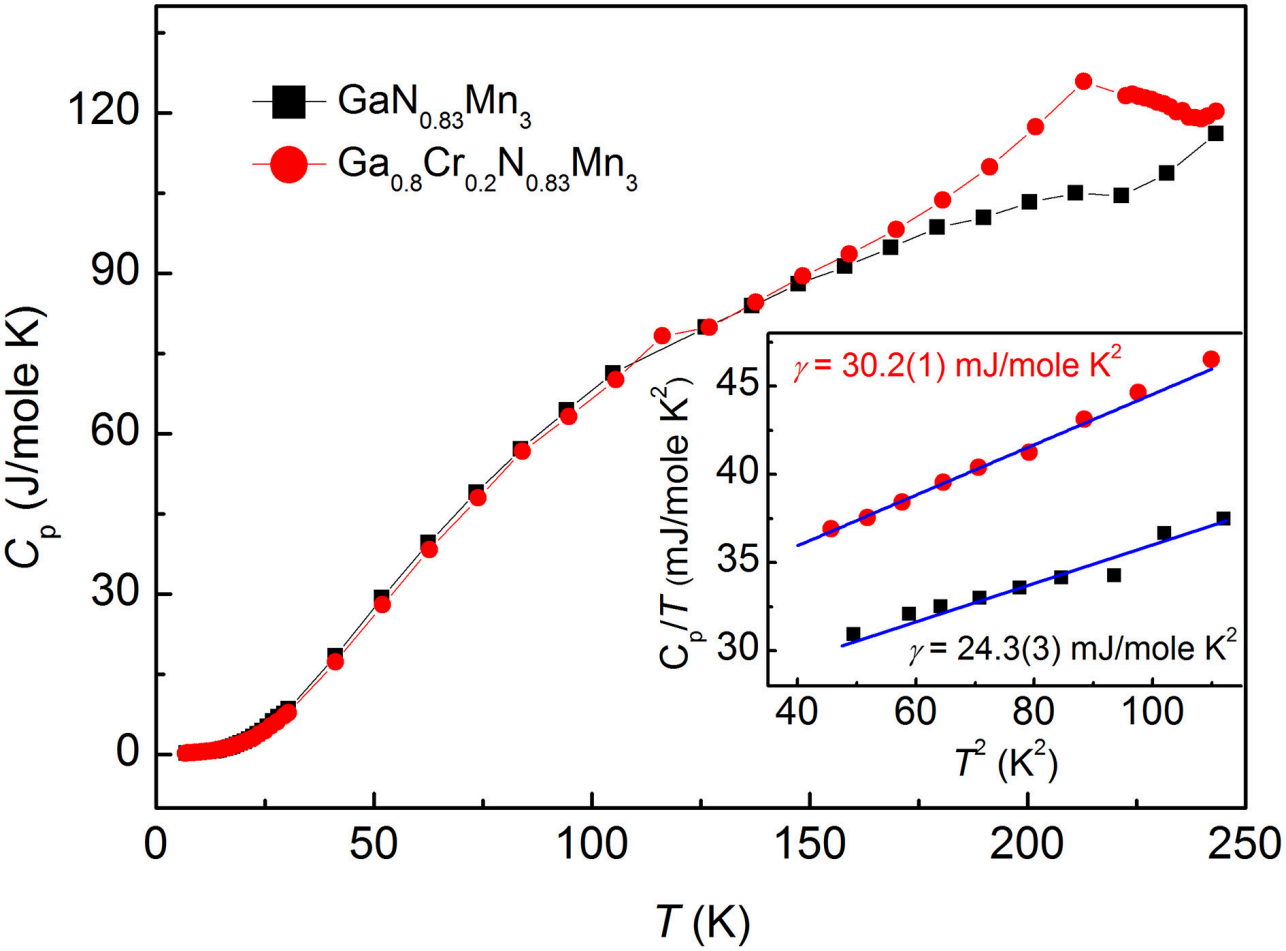
Specific heat *C*_*p*_(*T*) for Ga_1−*x*_Cr_*x*_N_0.83_Mn_3_ with *x* = 0 and 0.2. Inset shows a linear fit to the *C*_*p*_(*T*)/*T* vs. *T*^2^ curves at low temperatures. The fitted electronic coefficients of specific heat (γ, the Sommerfeld constant) are shown for both compounds.

Figure 5 shows the specific heat *C*_*p*_(*T*) for GaN_0.83_Mn_3_ and Ga_0.8_Cr_0.2_N_0.83_Mn_3_ between 6 and 245 K. A broad peak was observed at 220 K for *x* = 0.2 compound, which is resulted from the structural transition (i.e., the NTE) observed in Figure 4. As shown in the inset of Figure 5, the low-temperature specific heat data for each compound plotted as *C*_*p*_(*T*)/*T* vs. *T*^2^ can be well-fitted linearly by using the expression, *C*_*p*_(*T*)/*T* = γ+β*T*^2^, where γ (i.e., the Sommerfeld constant) represents for the electronic contribution, the second term is the lattice contribution based on the Debye approximation (Wang et al., 2010). The fitted values of γ are equal to 24.3(3) and 30.2(1) mJ/(mol K^2^) for GaN_0.83_Mn_3_ and Ga_0.8_Cr_0.2_N_0.83_Mn_3_, respectively. The value of γ corresponds to the density of the electronic DOS at E_F_ in the ground state. The enhanced γ in the Cr-doped compound indicates an enhancement of DOS at E_F_. According the Stoner criterion, FM interactions are enhanced in Cr-doped compound compared with the parent compound (Wang et al., 2010). This may explain why Cr doping suppresses the AFM ground state and finally changes the background to FM in *x* = 0.3 compound. According to the result reported by Garica, the γ is remarkably suppressed when the PM state transforms to AFM phase in GaNMn_3_ (Garcia et al., 1980), which suggests again that the increased DOS at E_F_ is not beneficial to the stabilization of AFM ground state of GaN_0.83_Mn_3_.

Although there are no theoretical reports on the electronic structure of Cr-doped GaNMn_3_, studies on GaNMn_3_ and Mn_4_N may give some hints of understanding the magnetism of current compounds. In GaNMn_3_, all Mn atoms locate at the face centers of the cubic lattice. The hybridized Mn 3d states with N 2p orbitals contribute mainly to the DOS at E_F_ (Miao et al., 2005). But Ga contributes little to the overall DOS at E_F_ (Miao et al., 2005). However, as to Mn_4_N, the corner Mn atoms (MnI) contribute a lot to the DOS at E_F_, while contribution from the face-center Mn atoms (MnII) is very similar to that in GaNMn_3_ (Miao et al., 2005). In Mn_4_N the magnetic moments at MnI (3.5 μB) are antiparallel to those at MnII (0.9 μB), leading to a ferrimagnetic ground state below 756 K (Takei et al., 1962). So MnI atoms play a dominant role in determining the magnetic properties of Mn_4_N. Analogously, when Cr elements occupy the corner sites (i.e., Ga sites) of the GaN_0.83_Mn_3_, their 3d orbitals will contribute to the DOS at E_F_. So the substitution of Cr for Ga introduces extra d electrons to the system, and thus increases the DOS at E_F_, leading to the enhanced FM interactions. A thorough theoretical study on the electronic band structures is needed in order to shed lights on the differences of magnetism and thermal expansion between Cr and Mn doped compounds.

## Conclusions

In summary, we report large NTE at low temperatures in antiperovskite compounds Ga_1−*x*_Cr_*x*_N_0.83_Mn_3_ (0 ≤ *x* ≤ 0.3). With increasing *x*, the NTE window was expanded and moved to lower temperatures quickly. For *x* = 0.1 and *x* = 0.2, the NTE occurs at 256–318 K (Δ*T* = 62 K) and 151–232 K (Δ*T* = 81 K) with an average α_*L*_ of −46 and −22.6 ppm/K, respectively. Finally, for *x* ≥ 0.3, NTE was not observed down to 5 K. As revealed by the specific heat measurement, Cr doping increases the DOS at E_F_, which favors the emergence of FM order against the AFM background. The competing FM order was suggested to suppress the original AFM order and hinder its propagation upon cooling, leading to the NTE with wide Δ*T*.

## Author contributions

XG and PT designed the synthetic work; XG carried out the synthesis and characterization of all the compounds; JL, CY, KZ, and SL participated in characterization of structure and magnetism; WS and XG carried out the heat capacity; XG and PT analyzed the data and wrote the manuscript; PT revised the paper; YS did discussion for this work. All authors listed, have made substantial, direct, and intellectual contribution to the work, and approved it for publication.

### Conflict of interest statement

The authors declare that the research was conducted in the absence of any commercial or financial relationships that could be construed as a potential conflict of interest.
